# Neuropsychiatric Phenotype in a Patient with Neurodevelopmental Disorder with or Without Early-Onset Generalized Epilepsy (NEDEGE)

**DOI:** 10.3390/genes17060713

**Published:** 2026-06-21

**Authors:** Dominika Szczęśniak, Anna Wilczek, Magdalena Mroczek

**Affiliations:** 1 Department of Genetics, Institute of Psychiatry and Neurology, 02-957 Warsaw, Poland; dszczesniak@ipin.edu.pl; 2 Medgen Medical Center, 02-954 Warsaw, Poland; 3 Faculty of Medicine, Medical University of Warsaw, 02-091 Warsaw, Poland; anulawilczek@gmail.com; 4 Department of Biomedicine, University Hospital Basel, University of Basel, 4031 Basel, Switzerland; 5 Department of Consultation-Liaison-Psychiatry and Psychosomatic Medicine, University Hospital Zurich, University of Zurich, 8091 Zurich, Switzerland

**Keywords:** neurodevelopmental disorder with or without early-onset generalized epilepsy, NEDEGE, neuropsychiatry, rare diseases

## Abstract

We report the oldest female identified to date with a pathogenic *NBEA* variant who has been followed longitudinally. She presented with a complex, diagnostically inconclusive psychiatric phenotype extending in adulthood and a suspected mild neurodevelopmental impairment. The 64-year-old patient experienced recurrent episodes of mental state decompensation characterized predominantly by persecutory and health-related delusional ideation and anxiety. Her most recent psychiatric diagnosis was mixed conversion disorder. Although she never underwent formal cognitive testing, mild intellectual disability was suspected based on her educational attainment, occupational history, and social functioning. Additionally, the patient presented with a likely coincidental tremor. A history of childhood epilepsy could not be confirmed, as detailed epilepsy records were unavailable. Furthermore, the patient declined neuroimaging, precluding assessment of a possible relationship with the identified *EXT2* deletion. This case expands the currently recognized neuropsychiatric spectrum possibly associated with pathogenic *NBEA* variants, highlights the importance of extending phenotypic characterization in later adulthood, and underscores the value of longitudinal follow-up.

## 1. Introduction

Neurodevelopmental disorder with or without early-onset generalized epilepsy (NEDEGE; MIM 619157) is a dominantly inherited disease. *NBEA* is a haploinsufficiency gene with nonsense, frameshift, splice site, deletions and multigene deletion variants associated with NEDEGE phenotype [1]. *NBEA* encodes neurobeachin, a brain-specific anchoring kinase. *NBEA* was first described as a candidate gene for neurodevelopmental disorders (NDD) in two individuals in the NDD cohort in 2017 [2]. In 2018 a large cohort, 24 patients with de novo *NBEA* variants were reported [3]. All patients had NDD and speech delay, with two being nonverbal at the ages of 11 and 19. Most patients had also epilepsy with predominantly generalized childhood onset seizures, a subset had myoclonic astatic epilepsy (5/24), and two presented with developmental regression with seizure onset [3]. Half of the individuals have autism or prominent autistic features, in a few cases an aggressive behavior was reported. Since then, several case reports of patients with pathogenic *NBEA* variants have been published [4] and the phenotype has been broadened with dystonic features [5] and a possible association with brain malformations was discussed (*CAMSAP1* and *NBEA* overlapping variants in one patient) [6].

Mechanistically, *NBEA* encodes neurobeachin, a brain-enriched multidomain scaffolding protein involved in vesicle trafficking localizing near the Golgi apparatus [1,7]. Nbea is an important player in the functioning of inhibitory GABA synapses. Neurotransmitter GABA mediates removal of the GABA receptor (GABA_A_Rs). Nbea interacts with gephyrine that together with GABA_A_Rs condition inhibitory long-term potentiation of the GABA synapses [8]. Nbea is recruited to GABAergic synapses and regulates GABA_A receptor trafficking in a PKA-dependent manner, providing a plausible link between *NBEA* haploinsufficiency, altered excitation–inhibition balance, epilepsy, and neuropsychiatric manifestations [8]. Modeling of the *NBEA* pathogenic variants in *C. elegans* showed to impact the intracellular transport of potassium channel in neurons [1]. Further, NBEA interacts physically with the NOTCH1, making a link with autism spectrum disorders plausible in the light of NOTCH central role in the neurodevelopment [9]. On the genome-wide level, variants in *NBEA* have been associated with migraine in bipolar disorder [10].

## 2. Materials and Methods

This study is a single-patient case report. Written informed consent for genetic testing and publication of anonymized clinical and genetic data was obtained from the patient.

The genomic DNA of patients was extracted from a whole blood sample using Prepito. The sequencing exome library was prepared according to Twist Exome Enrichment (Twist Bioscience, San Francisco, CA, USA). The enriched DNA libraries were sequenced by the Illumina NovaSeq 6000 instrument (Illumina, Inc., San Diego, CA, USA), 2 × 100 bp. All procedures for exome sequencing were conducted by CeGaT (Tübingen, Germany). Variants were called using HaplotypeCaller (GATK v4.2.6.1, FreeBayes v1.3.2) and named using Variant Effect Predictor (VEP109). The variants were classified according to ACMG recommendations. The presence of the variant in control populations was checked in 1000 Genomes and gnomAD (Broad Institute, Cambridge, MA, USA) databases. The in silico splicing analysis was performed using algorithms embedded in Alamut Visual Plus software v.2.0 (Sophia Genetics, Boston, MA, USA), i.e., SpliceSiteFinder-like, MaxEntScam, NNSPLICE and GeneSplicer and SpliceAI. The XHMMv1.0 algorithm and in-house scripts were used to search for small, rare, hemizygous and homozygous copy number variation (CNVs). All clinically relevant variants were confirmed through Sanger sequencing.

## 3. Results

### 3.1. Clinical Presentation

A female patient, born in 1962, was referred to a Genetic Counseling Clinic at the Institute Psychiatry and Neurology, Warsaw, Poland, for evaluation due to suspected mild intellectual impairment. She was born from the third pregnancy and third delivery; however, the course of pregnancy remains unknown. According to the patient’s history, a perinatal injury was suspected and was considered the cause of her delayed psychomotor development. The family history is unremarkable. The patient has two older siblings: a sister born in 1960, who is healthy and completed secondary education, and a brother who graduated from technical school and presents normal intellectual functioning. The patient’s daughter is also healthy. There is no known family history of intellectual disability or related disorders.

The patient has a documented history of psychiatric care since 1985. At that time, a diagnosis of paranoid schizophrenia (ICD-10: F20.0) was made; however, the clinical picture has been atypical, as she has never exhibited core negative symptoms, such as diminished emotional expression, alogia, avolition, anhedonia, or asociality. She was hospitalized multiple times in psychiatric departments (in 1988, 1991, 1993, 2002, and 2003), receiving various diagnoses over time, including paranoid schizophrenia, anxiety disorders, and organic mental disorders coexisting with intellectual disability. During hospitalization in 1991, mild intellectual disability was diagnosed. More recently, during her last psychiatric hospitalization, the discharge diagnoses included mild intellectual disability and mixed dissociative (conversion) disorders. Since 2007, the patient has remained under outpatient psychiatric care due to anxiety disorders. Over the following decades, she presented with recurrent episodes of mental state decompensation characterized predominantly by delusional symptoms and anxiety. She has been treated with extended-release quetiapine at a dose of 150 mg daily, with a good therapeutic response. In 2019, she received a diagnosis of mixed dissociative (conversion) disorder (ICD-10: F44.7), although this classification does not fully account for the longitudinal clinical presentation. Periodic mental state decompensations occurred predominantly when the patient discontinued the treatment on her own. Also, the current psychiatric decompensation occurred after the patient independently discontinued her psychiatric medication. At present, she presents mainly with persecutory and health-related delusional ideation, expressing fears that other people are negatively disposed toward her and that she is about to develop a serious illness. Based on the available clinical documentation, no clear episodic mood disorder was identified. She is described as friendly and socially engaged, without features suggestive of autism spectrum disorder, such as persistent deficits in social communication and social interaction, as well as restricted, repetitive patterns of behavior, interests, or activities. She was identified as having mild intellectual disability based on her social functioning but never underwent any formal testing. Despite suspected cognitive impairment, the patient is functionally independent. She lives alone, works in a sheltered workshop, and is engaged in handicraft activities.

The patient had possible childhood epilepsy by history. In pediatric period, the patient was reportedly treated with valproic acid, although no medical documentation is available. She has never undergone a formal assessment for epilepsy. Now, the patient is not receiving any antiepileptic treatment, remains seizure-free, and an EEG performed around the age of 60 was normal. At the age of 59, she was diagnosed with tremor, which responded well to propranolol treatment indicating the diagnosis of essential tremor.

Additional investigations included a normal EMG. A CT scan of the central nervous system revealed mild symmetrical calcifications, while the patient declined MRI. Her medical history is also notable for arterial hypertension, chronic open-angle glaucoma, status post bilateral cataract surgery, status post cholecystectomy, and hemorrhoids.

### 3.2. Physical Examination

The patient presented with short stature (height of 152 cm) and a normal occipitofrontal circumference. Her body proportions were symmetrical. Dysmorphic features included a high forehead with frontal bossing. Notably, on palpation, there were bilateral, hard, non-tender bony thickenings located in the distal metaphyseal regions of the forearm bones and wrist joints. These lesions were immobile relative to both the underlying structures and the overlying skin. The skin covering the lesions appeared normal, with no signs of inflammation. In the absence of imaging studies, the exact nature of these lesions cannot be definitively established.

### 3.3. Genetic Testing

Cytogenetic analysis revealed a normal female karyotype (46,XX). Array comparative genomic hybridization (aCGH) identified a deletion involving exon 13 of the *EXT2* gene. The detected *EXT2* alteration involves a heterozygous deletion encompassing exon 13 and adjacent intronic regions. As the exact breakpoints could not be determined by the applied methods (aCGH, MLPA, and WES-based). This finding was confirmed through whole-exome sequencing with CNV analysis. Additionally, whole-exome sequencing detected a heterozygous frameshift variant in the *NBEA* gene, NM_015678.4(NBEA):c.43_44del (p.Gln15GlyfsTer56). This is a novel heterozygous frameshift variant that has not been reported in population databases. The variant is predicted to result in loss of normal protein function, which is a well-established disease mechanism for *NBEA*-related disorders. According to ACMG/AMP guidelines, the variant was classified as likely pathogenic based on the criteria PVS1 and PM2.

## 4. Discussion

We report a female with a complex psychiatric phenotype in addition to the typical features of NEDEGE. A notable aspect of the present case is the longitudinal adult observation. Most individuals with pathogenic *NBEA* variants have been described in pediatric cohorts [3], and data on adult neuropsychiatric trajectories [5,11] remain limited. To our knowledge, this patient represents one of the oldest individuals reported with a pathogenic *NBEA* variant. This case, therefore, contributes to the limited body of evidence on the natural history and possible long-term neuropsychiatric manifestations associated with *NBEA* haploinsufficiency.

Overall, our patient presented with complex psychiatric symptomatology. The patient’s psychopathological profile does not meet the clear diagnostic criteria for any single disorder as defined by ICD-10 and remains diagnostically inconclusive within current nosological frameworks. Initially, a diagnosis of paranoid schizophrenia (ICD-10: F20.0) was made; however, the patient did not fulfill the ICD-10 diagnostic criteria, as she exhibited neither any of the symptoms from group 1 nor at least two symptoms from group 2 [12]. She presented with persecutory and health-related delusional ideation. No delusions of control or influence, delusional perception, persistent hallucinations, or negative symptoms were described. Furthermore, the onset or exacerbation of symptoms was not temporally associated with psychosocial stressors and appeared after quetiapine treatment was discontinued. The symptom profile was also not typical of mixed dissociative (conversion) disorder (ICD-10: F44.7), particularly as anxiety was one of the predominant symptoms. The long-standing and chronic nature of psychiatric symptoms argues against a late-life onset psychiatric disorder. Cognitive impairment/intellectual disability had already been recognized decades earlier in the clinical course.

To our best knowledge, just one case with a psychiatric diagnosis in a patient having a variant in the *NBEA* gene has been reported [11]. A 27-year-old male with a 13q12 deletion including *NBEA* gene was diagnosed with autism spectrum disorder, obsessive–compulsive disorder, and schizophrenia. He presented with significant decline in executive and occupational function over the last year, social withdrwal, poverty of speech, echolalia and perseveration. The previous treatment with escitalopram 20 mg, lamotrigine 200 mg, and ziprasidone 40 mg became ineffective over the last 5 years, but he was successfully treated with prazosin [11]. Available data suggest behavioral disturbances such as aggression and attention deficits in children with NEDEGE. In the largest coohort decribed so far, seven patients showed behavioral problems (4/24 aggression; 4/24 attention deficits/hyperactivity) [3]. However, structured psychiatric phenotyping is largely lacking [3] in both adult and children (Table 1). The recurrent delusional and anxiety-dominated decompensations observed in our patient may represent an underrecognized component of the adult *NBEA*-related phenotype, although causality cannot be established based on a single case.

We further note that a possible link between NBEA, the glutamatergic transmission system, epilepsy, and psychiatric symptoms warrants further attention. Patients with GluR antibodies have been reported to show markedly impaired psychosocial functioning, including learning difficulties and psychiatric symptoms, as well as peri- and ictal psychosis with delusions and anxiety [13]. A proportion of such epilepsy cases may remain undiagnosed. However, in our patient, the psychotic episodes appeared to be more closely related to discontinuation of medication than to other diagnoses.

Movement disorders have been recognized within the expanding phenotypic spectrum of NEDEGE. From the largest cohort of 24 patients described so far, eight had movement abnormalities (6/24 wide-based, uncoordinated gait; 3/24 dystonia; 1 clumsiness) [3]. In one adult patient dystonia was reported [5]. However, essential tremor has not been established as a recurrent manifestation. Given the late onset and good response to propranolol in our patient, the tremor is more likely coincidental rather than directly related to the *NBEA* pathogenic variant.

The in silico prediction of the *NBEA* variant’s pathogenicity provides further insight into its possible molecular mechanism. Although the variant introduces a premature termination codon, it is located close to the translation initiation site (<100 nucleotides from the start codon) and is therefore not expected to trigger nonsense-mediated mRNA decay. Instead, it is predicted to produce an extremely truncated protein (p.Gln15GlyfsTer56) lacking virtually all known functional domains of NBEA. Furthermore, numerous pathogenic loss-of-function variants, including frameshift and nonsense variants, have been reported throughout the gene, supporting haploinsufficiency as the established disease mechanism.

Another genetic finding that should be taken into account, although not related to *NBEA* haploinsufficiency, is a deletion of the *EXT2* gene. Pathogenic or likely pathogenic variants in *EXT2* are associated with hereditary multiple exostoses (MIM 133701), an autosomal dominant disorder. Exostoses are usually localized in the long bones of the lower or upper limbs and the thorax [14]. However, in the context of our patient, bony thickenings, although not confirmed radiologically, should not be interpreted as hereditary multiple osteochondromas and may represent incidental findings. Importantly, even if confirmed, these skeletal findings would be considered independent of the *NBEA*-related neurodevelopmental phenotype.

As a limitation of this report, the lack of detailed imaging and formal neuropsychological assessment, uncertainties regarding the epilepsy diagnosis, limitations related to neuropsychiatric phenotyping, and the lack of familial segregation analysis represent important constraints of this study. The patient declined brain MRI and radiological bone imaging. MRI findings in patients with NEDEGE usually do not show specific features; however, further imaging would have been important to elucidate the etiology of the calcifications observed on CT in the context of neuropsychiatric features. These calcifications may have different etiologies, including age-related, metabolic, vascular, medication-related, or may be unrelated to *NBEA* variant. The lack of radiological confirmation also has implications for the interpretation of the *EXT2* deletion. In this context, we cannot interpret bony thickening as osteochondromas and therefore the *EXT2* deletion cannot be interpreted as disease-causing. With regard to neuropsychological assessment, the patient is currently over 60 years of age, and historical medical and psychological records from childhood were not available for review. Therefore, the basis of the original diagnosis could not be verified retrospectively. Nevertheless, a history of lifelong cognitive impairment was supported by the patient’s educational trajectory, including attendance at a special education school and subsequent vocational training in a specialized institution for individuals with intellectual disabilities. The diagnosis of epilepsy is also uncertain. Although the patient was treated, no epilepsy-specific diagnostic workup was available in the records, and it is not possible to determine on what basis the diagnosis was made. Given that the current EEG is normal and there is no history of seizures, the diagnosis of epilepsy should be interpreted with caution. There are further uncertainties regarding the neuropsychiatric phenotype. The patient underwent multiple hospitalizations; however, detailed discharge letters from these admissions were not available. Inconsistencies in clinical descriptions may reflect the complexity of the psychiatric presentation and may have contributed to changes in diagnostic attribution over time. Genetic testing results were not available for the patient’s parents or siblings. The patient’s parents were deceased at the time of the study, and she had no contact with her siblings, precluding further family investigations. However, genetic testing was performed in the patient’s daughter, who does not have intellectual disability and was found not to carry the *NBEA* variant identified in the proband. Although this finding does not establish a de novo occurrence, it is consistent with the potential pathogenicity of the variant. Overall, we acknowledge that, based on a single case report and the rarity of the condition, a causal relationship cannot be established, and incidental or concurrent findings must be considered.

In summary, we report an adult female with a pathogenic *NBEA* variant, mild neurodevelopmental impairment, and a complex, diagnostically inconclusive psychiatric phenotype extending into adulthood. There could be a possible association of neuropsychiatric phenotype with pathogenic variants on *NBEA* therefore patients should be examined in this context.

## Figures and Tables

**Table 1:** Features in pediatric and adult patients and comparison with a reported case in patient with neurodevelopmental disorder with early-onset generalized epilepsy (NEDEGE).

Feature	Pediatric Cohorts (Literature)	Adult Cases (Literature)	Present Case
Age at diagnosis	Infancy/childhood [3]	Limited data [5,11]	60 years (one of the oldest reported cases)
Intellectual disability	Common (often moderate) [3]	Persists from childhood [5,11]	Mild (assessed based on history, e.g., educational trajectory; no formal standardized cognitive testing).
Speech delay	Very common [3]	Persists from childhood [11]	Present in childhood history.
Epilepsy	Frequent, often early-onset generalized [3]	Less consistently reported	Suspected in childhood, currently seizure-free
Autism spectrum features	Present in around 50% of the patients [3]	Unclear/underreported	Not observed
Psychiatric manifestations	Poorly characterized; behavioral disturbances reported, such as aggression (in 4 out of 24 patients) and attention deficits/hyperactivity (4 out of 24) [3]	Rare reports; one case described a 27-year-old male with ASD, obsessive–compulsive disorder and schizophrenia [11]	Prominent in the clinical picture: recurrent delusional episodes, anxiety disorders, diagnosis of mixed dissociative (conversion) disorder, and a chronic atypical schizophrenia presentation
Movement disorders	Observed in some cases, e.g., wide-based and uncoordinated gait, clumsiness (in 7 out of 24 patients) [3]	Emerging data indicate dystonia among others [5]	Essential tremor diagnosed at age 59, with a good response to propranolol (considered a likely incidental finding unrelated to *NBEA* variant).
Neuroimaging	Variable abnormalities [3]	Scarce data [5]	Head CT revealed mild symmetrical calcifications; the patient declined MRI scan

## Data Availability

The data presented in this study are available on request from the corresponding author.

## References

[1] Boulin T., Itani O., El Mouridi S., Leclercq-Blondel A., Gendrel M., Macnamara E., Soldatos A., Murphy J.L., Gorman M.P., Lindsey A. (2021). Functional analysis of a de novo variant in the neurodevelopment and generalized epilepsy disease gene NBEA. Mol. Genet. Metab..

[2] Bowling K.M., Thompson M.L., Amaral M.D., Finnila C.R., Hiatt S.M., Engel K.L., Cochran J.N., Brothers K.B., East K.M., Gray D.E. (2017). Genomic diagnosis for children with intellectual disability and/or developmental delay. Genome Med..

[3] Mulhern M.S., Stumpel C., Stong N., Brunner H.G., Bier L., Lippa N., Riviello J., Rouhl R.P.W., Kempers M., Pfundt R. (2018). NBEA: Developmental disease gene with early generalized epilepsy phenotypes. Ann. Neurol..

[4] Huang X., Wu W.L., Song J., Tian Y., Zhou Y., Wei S., Yu B., Qin L., Yang S. (2025). NBEA gene variant in a child with developmental disorder and epilepsy: A case report. Front. Neurosci..

[5] Schinwelski M., Krygier M., Sitek E.J., Mazurkiewicz-Bełdzińska M., Zech M. (2025). New Progressive Generalized Dystonia Phenotype in a Patient with NBEA-Related Neurodevelopmental Disease. Mov. Disord. Clin. Pract..

[6] Karatas E., Gulec A., Korkmaz M., Karaman Z.F., Kiraz A., Per H., Dundar M. (2024). Brain malformation, neurodevelopmental disorder and epilepsy in a case of two rare genetic diseases: Overlapping phenotype. Neurogenetics.

[7] Medrihan L., Rohlmann A., Fairless R., Andrae J., Döring M., Missler M., Zhang W., Kilimann M.W. (2009). Neurobeachin, a protein implicated in membrane protein traffic and autism, is required for the formation and functioning of central synapses. J. Physiol..

[8] Lützenkirchen F.P., Zhu Y., Maric H.M., Boeck D.S., Gromova K.V., Kneussel M. (2024). Neurobeachin regulates receptor downscaling at GABAergic inhibitory synapses in a protein kinase A-dependent manner. Commun. Biol..

[9] Tuand K., Stijnen P., Volders K., Declercq J., Nuytens K., Meulemans S., Creemers J. (2016). Nuclear Localization of the Autism Candidate Gene Neurobeachin and Functional Interaction with the NOTCH1 Intracellular Domain Indicate a Role in Regulating Transcription. PLoS ONE.

[10] Jacobsen K.K., Nievergelt C.M., Zayats T., Greenwood T.A., Anttila V., Akiskal H.S., BiGS Consortium, IHG Consortium, Haavik J., Bernt Fasmer O. (2015). Genome wide association study identifies variants in NBEA associated with migraine in bipolar disorder. J. Affect. Disord..

[11] Cantwell C.Y., Fortman J., Seegan A. (2021). Prazosin use in a patient with rare Neurobeachin gene deletion shows improvement in paranoid behavior: A case report. J. Med. Case Rep..

[12] Nickl-Jockschat T., Steiner J., Hirjak D., Hasan A. (2025). Schizophrenia and catatonia: From ICD-10 to ICD-11. Der Nervenarzt.

[13] Taiwo R.O., Goldberg H.S., Ilouz N., Singh P.K., Shekh-Ahmad T., Levite M. (2025). Enigmatic intractable Epilepsy patients have antibodies that bind glutamate receptor peptides, kill neurons, damage the brain, and cause Generalized Tonic Clonic Seizures. J. Neural Transm..

[14] Fusco C., Nardella G., Fischetto R., Copetti M., Petracca A., Annunziata F., Augello B., D’Asdia M.C., Petrucci S., Mattina T. (2019). Mutational spectrum and clinical signatures in 114 families with hereditary multiple osteochondromas: Insights into molecular properties of selected exostosin variants. Hum. Mol. Genet..

